# Assessment of gut-brain interactions: reframing DGBI symptoms from visceral hypersensitivity to computational interoceptive overfitting

**DOI:** 10.3389/fphys.2026.1817832

**Published:** 2026-04-15

**Authors:** Dakai Zeng, He Zeng, Zi Lin, Wen-Jing Yan

**Affiliations:** 1 Third Affiliated Hospital of Wenzhou Medical University, Wenzhou, China; 2 The Affiliated Kangning Hospital of Wenzhou Medical University, Wenzhou, China; 3 Zhejiang Provincial Clinical Research Center for Mental Health, The Affiliated Wenzhou Kangning Hospital, Wenzhou Medical University, Wenzhou, China

**Keywords:** body surface gastric mapping, digital therapeutics, disorders of gut-brain interaction, interoceptive overfitting, precision weighting

## Abstract

For decades, disorders of gut-brain interaction (DGBI) have been ensnared in an epistemological bottleneck, clinically managed as diagnoses of exclusion despite the absence of structural pathology on conventional endoscopy. Traditional bottom-up models of visceral hypersensitivity fail to explain the profound subjective-objective symptom mismatches observed in clinical practice. This Perspective proposes a radical paradigm shift: leveraging the Predictive Processing (PP) framework to reconceptualize DGBI as a hierarchical computational dysfunction termed “interoceptive overfitting”. We postulate that rigid, high-precision threat priors force the salience network (dACC and aIns) to misallocate pathologically high precision weighting to baseline physiological noise, such as healthy 3-cycles-per-minute (cpm) gastric slow waves. This top-down failure synthesizes illusory pain and triggers genuine autonomic disruption via active inference, creating a self-fulfilling loop of GI micro-sabotage. We present a clinical roadmap utilizing high-resolution body surface gastric mapping (BSGM) and Ecological Momentary Assessment (EMA) to identify “Probabilistic Mismatch Points” within a multimodal diagnostic matrix that accounts for non-rhythmic peripheral modulators. To resolve therapeutic stagnation, we propose closed-loop digital therapeutics (DTx) designed to recalibrate the brain’s predictive engine through validation-correction loops, targeted extinction learning, and dual-stream telemetry. This computational framework provides a rigorously scientific blueprint to resolve therapeutic stagnation in DGBI.

## 1 Introduction

Disorders of gut-brain interaction (DGBI), encompassing highly prevalent conditions such as functional dyspepsia (FD) and irritable bowel syndrome (IBS), affect an estimated 40% of the global population across their lifespan, posing a staggering socioeconomic and healthcare burden (Sperber et al., 2021). While the Rome IV criteria successfully standardized the clinical classification of these symptom clusters—shifting the nomenclature from the stigmatizing “functional” label to a bidirectional gut-brain axis model (Drossman, 2016)—DGBI diagnoses remain intrinsically apophatic. The persistent absence of mechanistically-driven biomarkers not only traps patients in costly, protracted diagnostic odysseys but also perpetuates the psychosocial stigma that these conditions are purely “psychosomatic” or “all in the head” (Black et al., 2020; Keefer et al., 2022).

In the daily clinical practice of a gastrointestinal endoscopist, this diagnostic ambiguity is a recurring frustration. We routinely perform exhaustive structural evaluations—such as esophagogastroduodenoscopies (EGD) and colonoscopies—only to observe visually pristine, perfectly healthy mucosa in patients reporting agonizing abdominal pain, severe nausea, or early satiety. This direct visual confirmation of diagnostic ambiguity exposes a fundamental limitation in the underlying pathophysiological models that have historically dominated gastroenterology. For decades, the field has been anchored to a “bottom-up,” structuralist perspective, tirelessly seeking the genesis of symptoms in peripheral end-organ abnormalities—such as delayed gastric emptying, impaired fundic accommodation, or low-grade mucosal inflammation (Ford et al., 2020).

Robust evidence demonstrates that peripheral gastrointestinal (GI) motility metrics correlate extremely poorly with the severity of patient-reported symptoms. For instance, patients with profound diabetic gastroparesis often report minimal discomfort despite objectively delayed gastric emptying; conversely, FD patients presenting with agonizing pain, severe nausea, or early satiety frequently exhibit entirely normal gastromotor and myoelectrical profiles (Carson et al., 2021). This “motility-perception decoupling” strongly implies that the core pathological hub dictating DGBI symptom severity does not reside at the site of peripheral signal generation. Instead, it is embedded within the central computational networks of the gut-brain axis—specifically, in how afferent interoceptive signals are filtered, integrated, and algorithmically decoded by the brain (Mayer et al., 2022). To overcome the current translational impasse, gastroenterology urgently requires a conceptual paradigm shift.

## 2 Conceptual reshaping: from linear visceral hypersensitivity to algorithmic dysfunction

To reconcile the subjective-objective mismatch, the field previously introduced the concept of “visceral hypersensitivity,” positing that peripheral afferent neurons or spinal dorsal horn pathways become sensitized, thereby amplifying normal physiological stimuli (Simrén et al., 2018). While contemporary iterations of the visceral hypersensitivity model acknowledge ‘central sensitization,’ the framework often lacks a precise mechanistic explanation for how and why normal signals are amplified in specific contexts. It implicitly relies on a linear amplification metaphor. In contrast, the predictive processing framework does not just amplify a signal; it actively synthesizes perception based on prior expectations, explaining the profound context-dependency of symptoms. Consequently, it fails to explain the profound context-dependency of DGBI symptoms: why debilitating flare-ups can be triggered entirely by anticipatory anxiety or traumatic recall, and conversely, why symptoms can vanish instantaneously during states of deep cognitive absorption or targeted hypnotherapy (Kano et al., 2020).

**Figure 1:**
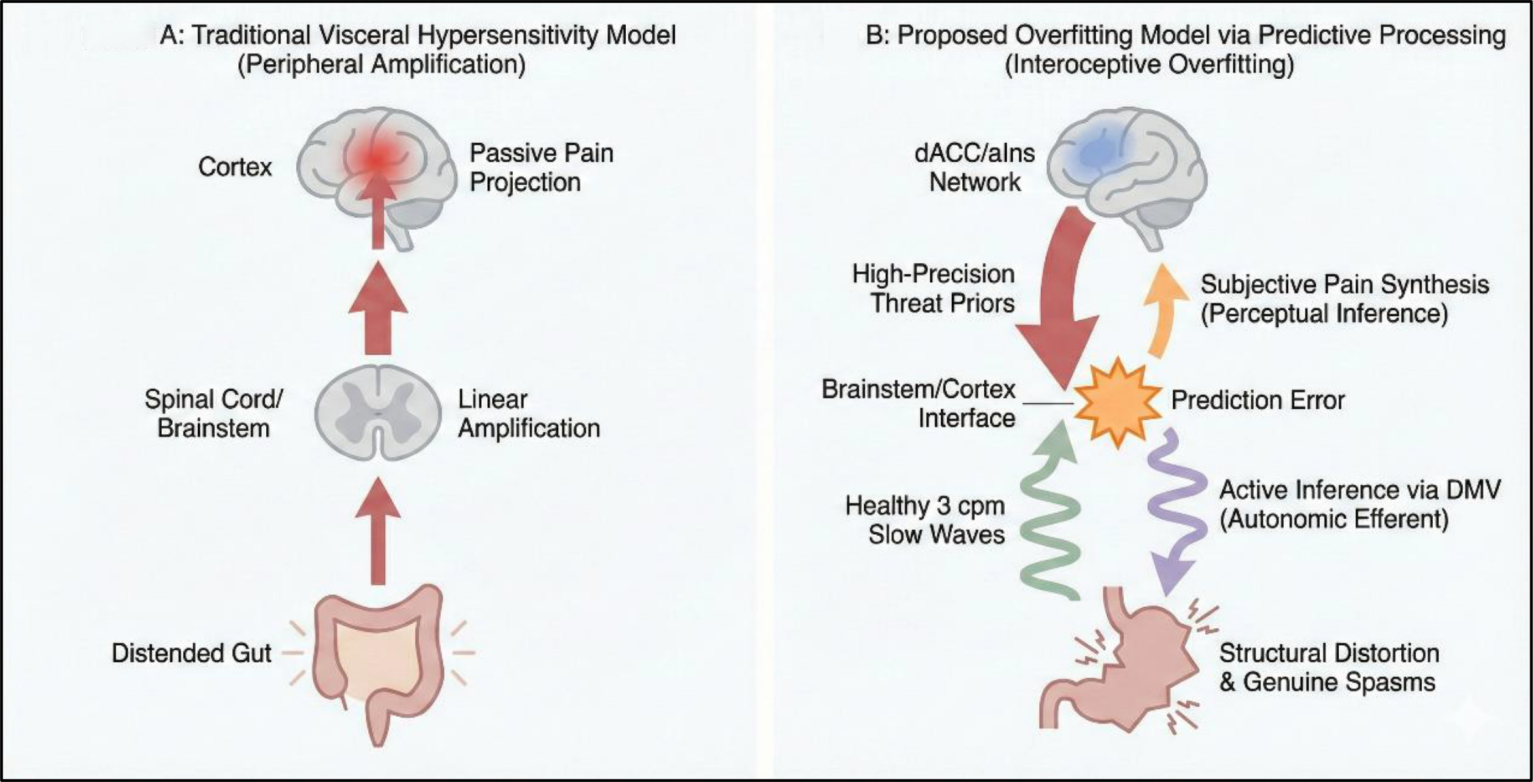
Comparison of DGBI pathophysiological models: From linear visceral hypersensitivity to interoceptive overfitting. Panel **(A)** illustrates the traditional bottom-up framework where peripheral signals undergo linear amplification within the spinal cord and brainstem. Panel **(B)** presents the proposed top-down model of “interoceptive overfitting,” where the salience network (dACC/aIns) assigns pathologically high precision weighting to rigid threat priors. This computational failure causes the brain to interpret healthy 3 cpm gastric slow waves as significant prediction errors, synthesizing subjective pain while triggering genuine autonomic disruption through active inference via the dorsal motor nucleus of the vagus (DMV). This descending “autonomic storm” results in structural GI micro-sabotage, creating a self-fulfilling pathological loop between computational errors and physical tissue distress.

Contemporary cognitive neuroscience has decisively abandoned the archaic notion of the brain as a passive receiver of somatic stimuli, replacing it with the Predictive Processing (PP) framework (Lee et al., 2021) (see **Figure 1**). According to PP, the brain operates as an active, forward-looking “Bayesian inference engine.” It continuously generates internal generative models (priors) regarding the internal and external state of the body to predict sensory inputs, constantly striving to maintain allostasis by minimizing “prediction errors” (PEs)—the mathematical discrepancy between the anticipated expectations and the actual afferent signals (Seth and Friston, 2016).

Within the computational framework of interoception (the central nervous system’s process of sensing and interpreting internal physiological signals) (Khalsa et al., 2018), the generation of subjective perception can be distilled into an elegant Bayesian heuristic (Barrett and Simmons, 2015):

(1) $$\text{Subjective Perception}\approx \mu_{\text{prior}}+\omega (y-\mu_{\text{prior}})$$

Where:

${\text{μ}}_{\text{prior}}$ (The Threat Prior): This represents the brain’s internal generative model or “prior expectations” regarding the state of the gut. In DGBI, these are often rigid, high-precision threat priors—essentially “expectations of pain”—arising from early-life adversity or post-infectious trauma.

ω (Precision Weighting): Acting as a computational rheostat, this variable represents the “reliability” or signal-to-noise ratio assigned to incoming sensory data. This is neuroanatomically orchestrated by the dorsal anterior cingulate cortex (dACC) and anterior insula (aIns).

$\left(\text{y}{\text{μ}}_{\text{prior}}\right)$ (The Prediction Error, PE): This is the mathematical discrepancy between the actual afferent signals (y), such as healthy 3 cpm gastric slow waves, and the anticipated threat ( ${\text{μ}}_{\text{prior}}$).

To move beyond a heuristic, we define the generative process where the brain estimates the hidden state of the gut (*s*) based on noisy afferent observations (*y*). In this Bayesian framework, the Precision Weighting ( ω) is formally the inverse of the expected sensory variance ( $\text{ω}=1/{\text{σ}}^{2}$). The subjective perception of pain (*P*) is thus the posterior belief, calculated as the precision-weighted combination of the threat prior ( ${\text{μ}}_{\text{prior}}$) and the sensory input:

(2) $$P=\frac{\omega_{prior}\mu_{prior}+\omega_{sensory}y}{\omega_{prior}+\omega_{sensory}}$$

In DGBI, the pathology is an over-representation of ωsensory relative to the actual signal-to-noise ratio of the gut, forcing the brain to ‘fit’ every minor fluctuations in the 3 cpm slow wave to a threat-based model. To resolve the apparent paradox between high-precision priors and high sensory weighting, we propose a hierarchical interpretation. In interoceptive overfitting, the ‘rigidity’ of a top-down threat prior (arising from trauma or infection) necessitates a corresponding pathological increase in the precision weighting ( $\omega_{sensory}$) assigned to bottom-up noise. Because the high-level generative model cannot be ‘falsified’ by the reality of a healthy gut, the dACC/aIns network compensates by ‘searching’ for evidence within benign 3 cpm fluctuations, erroneously elevating the signal-to-noise ratio of background noise to satisfy the high-precision expectation of pain. This captures the true nature of the ‘overfitting’ metaphor: the brain builds a hyper-complex model that interprets every minor physiological ripple as a significant confirmational data point for pathology.

## 3 “Interoceptive overfitting” and the descending pathological loop

### 3.1 The neuroanatomical basis of precision weighting and the “overfitting” metaphor

In the predictive processing equation, Precision Weighting acts as the critical rheostat governing systemic allostatic stability. It represents the brain’s statistical estimation of the reliability or “signal-to-noise ratio” of a specific sensory input (Petzschner et al., 2021). Neuroanatomically, this computation is orchestrated by the brain’s salience network: the anterior insula (aIns) maps and represents interoceptive prediction errors, while the dorsal anterior cingulate cortex (dACC) evaluates the threat imminence of these errors and dynamically allocates precision weighting (Uddin, 2015; Khalsa et al., 2018).

In a healthy state, the basal 3 cpm electrical slow waves generated by the interstitial cells of Cajal (ICC) in the stomach are evaluated by the dACC as predictable, low-value background noise (Rebollo et al., 2021). Consequently, they are assigned extremely low precision weighting and are effectively filtered out before reaching conscious awareness.

Here, we propose the central mechanistic hypothesis of this Perspective: The pathogenesis of DGBI is characterized by a failure in Bayesian precision-weighting, where the internal model exhibits a biological form of ‘overfitting.’ Rather than failing to generalize, the brain’s generative model becomes so hyper-precise in its threat expectations that it interprets stochastic physiological fluctuations as meaningful signals of pathology.

Due to early-life adversity, acute GI infections (e.g., post-infectious IBS), or chronic allostatic load, the patient’s interoceptive network becomes infected with rigid, high-precision threat priors (Smith et al., 2021). Under this algorithmic dysfunction, the dACC erroneously allocates disproportionately massive precision weights to mundane, benign visceral afferents. Even when the stomach exhibits perfectly healthy, non-pathological 3 cpm slow-wave perturbations, the “overfitted” brain latches onto this subtle physiological noise. Refusing to categorize it as a safe background state, the brain enforces a top-down fit, interpreting the noise as a massive prediction error indicating “tissue damage or extreme distension.” This algorithmic pattern-recognition failure translates directly within the aIns into the conscious experience of agonizing pain, nausea, or bloating (Smith et al., 2020).

It is critical to acknowledge that this ‘overfitting’ does not occur in a biological vacuum. Peripheral factors—such as low-grade mucosal inflammation or microbiome dysbiosis—likely act as ‘stochastic modulators’ (Hall et al., 2022). While these factors may not reach the threshold of structural pathology, they increase the entropy (volatility) of the visceral signal (y). For a brain already burdened by high-precision threat priors, this increased signal volatility provides more ‘data points’ for the dACC to erroneously categorize as pathology, thereby accelerating the overfitting loop.

### 3.2 Top-down autonomic disruption via active inference

Crucially, framing DGBI as a “computational dysfunction” does not imply that the patient’s symptoms are fabricated “psychogenic hallucinations.” Within the PP framework, to resolve massive prediction errors, the brain has two choices: update its priors (perceptual inference) or alter the physical state of the body to align with the prior—a process known as Active Inference (Pezzulo et al., 2015; Seth and Friston, 2016).

Driven by intractable threat predictions, descending top-down directives from the ventromedial prefrontal cortex (vmPFC) fail to inhibit limbic hyperreactivity (e.g., the amygdala). This failure triggers an excessive efferent outflow through the dorsal motor nucleus of the vagus (DMV) and sympathetic efferent pathways (Browning et al., 2017). Crucially, this top-down autonomic storm transitions the pathology from a purely computational error to a structural reality. By imposing genuine physical sabotage upon the GI microenvironment (e.g., inducing localized microvascular ischemia or secondary ICC dysrhythmias), the descending pathways generate actual nociceptive signals. This creates a tragic ‘self-fulfilling prophecy’: the brain’s initial algorithmic overfitting to benign noise eventually manifests the exact physical tissue distress it falsely predicted, thereby cementing a rigid, self-evidencing Computational-Physiological Pathological Loop. This elegantly explains why mild motility abnormalities or reduced heart rate variability (HRV) are sometimes observed in DGBI cohorts: they are often the downstream consequences of central predictive dysregulation, rather than the primary upstream etiology (Van den Bergh et al., 2017).

## 4 Objective anchors via high-resolution gastric mapping

To systematically dismantle the self-evidencing pathological loop of interoceptive overfitting, clinical researchers must first establish a reliable, objective coordinate system of the gastrointestinal “ground truth.” (Gharibans et al., 2022) Historically, attempts to measure gastric myoelectrical activity via legacy single-channel electrogastrography (EGG) failed to fulfill this role. Plagued by low spatial resolution, poor signal-to-noise ratios, and severe susceptibility to motion artifacts, traditional EGG was rendered largely obsolete for precision ambulatory phenotyping (Wang et al., 2024).

### 4.1 Body surface gastric mapping and flexible wearables

The advent of high-resolution Body Surface Gastric Mapping (BSGM) (e.g., the FDA-cleared Gastric Alimetry system) has fundamentally revolutionized non-invasive GI electrophysiology. Utilizing dense, conformal multi-electrode arrays (e.g., 64-channel grids) coupled with advanced artifact-rejection machine learning algorithms, modern BSGM maps the spatial propagation, amplitude, and temporal stability of gastric slow waves with unprecedented fidelity, effectively distinguishing genuine dysrhythmias from benign physiological noise (O’Grady et al., 2023).

Concurrently, breakthroughs in materials science—specifically the development of highly compliant, organic-inorganic nanocomposite sensors (e.g., stretchable PEDOT: PSS interfaces)—have catalyzed the emergence of flexible epidermal EGG (FlexEGG). These soft, wearable bio-adhesives maintain sub-ohm impedance stability even under significant epidermal strain (>20%) (Oldroyd et al., 2024). This electromechanical compliance enables the continuous, ambulatory capture of ultra-low-frequency (0.05–0.2 Hz) gastric pacemaking signals during real-world daily activities and stress induction (Xing et al., 2025). Within our proposed framework, these wearable devices continuously quantify the bottom-up sensory prior.

### 4.2 Identifying “computational mismatch points”

Establishing a neuro-computational diagnosis requires the temporal alignment of objective physiological data with the patient’s subjective state. Retrospective clinical questionnaires are profoundly skewed by recall bias and peak-end cognitive heuristics. To circumvent this, the modern diagnostic paradigm integrates continuous BSGM telemetry with Ecological Momentary Assessment (EMA). Delivered via smartphone interfaces, EMA prompts patients to dynamically log their symptom severity, bloating, and contextual anxiety in their natural environments (Chan et al., 2019).

By mathematically cross-registering these two data streams, clinicians can generate a bidimensional diagnostic coordinate system: the Mismatch Matrix. When algorithmic analysis identifies a temporal epoch where the underlying BSGM rhythm exhibits unperturbed homeostasis—characterized by high spatial stability and a normal 3.0 cpm principal frequency—yet the concurrent EMA registers an agonizing symptom spike, this divergence is defined as a ‘Probabilistic Mismatch Point’. While such points provide empirical evidence of interoceptive overfitting, clinicians must remain vigilant regarding electromechanical uncoupling. In these instances, the brain may be amplifying non-pathological background noise. Clinicians must interpret BSGM as a high-fidelity filter for rhythmic dysrhythmias rather than a binary indicator of health. To account for electromechanical uncoupling, the ‘Mismatch Matrix’ must incorporate mechanical compliance (e.g., via fundic impedance) and chemical signaling as latent variables. This ensures that a normal 3 cpm rhythm does not lead to a premature dismissal of genuine mechanical dysfunction, such as impaired fundic accommodation.

While BSGM provides a high-fidelity anchor for rhythmic assessment, it is not a binary indicator of peripheral health. To ensure diagnostic robustness, the ‘Mismatch Matrix’ must be interpreted within a multimodal context. When a ‘Probabilistic Mismatch Point’ is identified (normal rhythm vs. high symptoms), clinicians should treat this as a signal for potential interoceptive overfitting while simultaneously considering non-rhythmic ‘stochastic modulators’ such as low-grade inflammation or dysbiosis. Consequently, we propose that the Mismatch Matrix incorporate mechanical markers (e.g., fundic impedance) and chemical signaling as latent variables to definitively distinguish between computational overfitting and genuine, non-rhythmic peripheral dysfunction.

## 5 Closed-loop digital therapeutics

Once an interoceptive overfitting pattern is phenotyped, the therapeutic objective must radically pivot from “suppressing peripheral symptoms” (e.g., via antispasmodics) to “recalibrating the central predictive engine.” Closed-loop Digital Therapeutics (DTx) utilizing computational biofeedback represent the vanguard of this neurogastroenterological paradigm shift (Keefer et al., 2022).

### 5.1 Interoceptive exposure and extinction learning

Traditional cognitive-behavioral therapies (CBT) for DGBI often rely heavily on verbal reappraisal, which may fail to penetrate deeply entrenched, unconscious autonomic reflexes. A closed-loop DTx system, however, leverages real-time physiological telemetry as an incontrovertible feedback medium to facilitate targeted interoceptive exposure.

When the DTx algorithm detects a “Computational Mismatch Point”—the patient reports pain via EMA, but the wearable FlexEGG confirms a stable3 cpm rhythm—the system triggers immediate, visually salient positive feedback. For example, a smartphone dashboard might display a soothing visualization of the rhythmic gastric waves alongside a cognitive reappraisal prompt: “Your stomach rhythm is currently perfectly stable and safe. The distress you feel is a false alarm generated by neural amplification, not physical damage.”.

This precise, real-time juxtaposition forces the predictive brain to confront a massive exogenous prediction error: the subjective prediction of “threat” directly conflicts with the objective “ground truth” of safety. Repeated exposure to this discrepancy initiates extinction learning—a robust neuroplastic mechanism wherein the ventromedial prefrontal cortex (vmPFC) strengthens its top-down inhibitory control over the hyperactive anterior insula and amygdala, gradually disarming the pathological threat prior (Milad and Quirk, 2012).

### 5.2 Synaptic recalibration (regularization) of the predictive engine

From a computational perspective, this continuous exposure to disconfirming ground truth acts as a form of mathematical “regularization,” penalizing the overfitted neural network. Through iterative biofeedback sessions, the brain undergoes Bayesian updating, forcing the dorsal anterior cingulate cortex (dACC) to dynamically downgrade the “precision weighting” it assigns to routine visceral noise14. Consequently, benign gastric signals are relegated back to subconscious processing. Over time, this synaptic recalibration restores allostatic harmony across the gut-brain axis, permanently extinguishing both the subjective pain and the descending autonomic dysregulation (Jeanne et al., 2023).

## 6 Scientific boundaries, limitations, and future directions

While the computational neurogastroenterology framework offers a highly cohesive theoretical model, its clinical translation demands rigorous epistemological humility.

### 6.1 Electromechanical uncoupling

Researchers must explicitly acknowledge a fundamental physiological limitation: electromechanical uncoupling. The gastric electrical slow wave mapped by BSGM dictates the potential for smooth muscle contraction, but electrical rhythmicity does not universally guarantee mechanical efficacy. Severe mechanical dysfunction—such as localized vagal neuropathy, fundic accommodation failure, or pyloric outlet obstruction—can still occur despite a perfectly normal 3 cpm slow wave (Camilleri et al., 2022).

Therefore, in the DGBI diagnostic pathway, BSGM should not be viewed as a wholesale replacement for gastric emptying scintigraphy (GES) or antroduodenal manometry. In a rigorously designed real-world clinical workflow, the Mismatch Matrix must be integrated alongside structural evaluations; specifically, patients who exhibit high-confidence ‘Probabilistic Mismatch Points’ on BSGM/EMA should still undergo confirmatory mechanical testing, such as a standardized 4-hour GES or wireless motility capsule evaluation, to definitively rule out silent pyloric outflow obstructions or severe mechanical gastroparesis before being triaged exclusively to digital therapeutics (Abraham et al., 2026). Rather, its supreme value lies in serving as a baseline bio-rhythmic filter. If a patient exhibits profound spatial dysrhythmias on BSGM (e.g., retrograde propagation), peripheral neuromodulatory or prokinetic interventions remain the priority. Conversely, if high-resolution mapping confirms pristine electrical stability alongside severe symptoms, it provides unparalleled justification to pivot aggressively toward central neuromodulators and closed-loop DTx.

### 6.2 Temporal latency in biofeedback

A critical engineering hurdle in gastric biofeedback is temporal latency. Unlike cardiovascular signals (which provide feedback multiple times per second), gastric slow waves are inherently sluggish (~20 seconds per cycle). Providing instantaneous biofeedback for such low-frequency signals poses a challenge for classic operant conditioning protocols (Mathew et al., 2025).

To overcome the ~20-second latency of gastric slow waves, which typically hampers traditional operant conditioning, we propose a Dual-Stream Telemetry architecture. This design does not dilute the intervention into a general relaxation exercise; rather, it uses high-frequency autonomic proxies (e.g., HRV or skin conductance) as a temporal ‘fast-track’ for the ‘slow’ gastric pacing data. By providing instantaneous feedback on the autonomic state in phase with the gastric slow wave, the system provides the brain with the precise timing needed to recognize the relationship between its descending top-down commands and the peripheral gastric rhythm. This ‘fast-slow’ integration is essential for restoring the allostatic harmony of the gut-brain axis through targeted synaptic recalibration (Mathew et al., 2025).

### 6.3 Epistemic trust and prior rigidity

A significant barrier to closed-loop DTx is the epistemic rigidity of the patient’s threat priors. A central nervous system dominated by high-precision anxiety predictions may simply reject disconfirming visual feedback from a wearable device, interpreting the discrepancy as a ‘faulty sensor’ rather than updating its internal model. Therefore, raw biofeedback is insufficient (Tsai et al., 2024). To mitigate the risk of clinical invalidation, DTx interfaces must move beyond simple ‘false alarm’ alerts and instead employ a sophisticated Validation-Correction Loop. Before the system presents objective data from the wearable sensor, it must first utilize EMA to acknowledge the subjective intensity of the patient’s symptoms. This sequence ensures ‘epistemic alignment,’ signaling to the patient that their agony is recognized as a biological reality rather than being dismissed as imaginary. By validating the experience before introducing the physiological ‘Mismatch’ data, the interface reduces the likelihood of defensive ‘prior rigidity’ and creates the psychological safety necessary for the brain to update its predictive engine.

## 7 Conclusion

Disorders of gut-brain interaction can no longer be relegated to the ambiguous realm of medically unexplained symptoms. By adopting the predictive processing framework, we can demystify the core pathology of these conditions: an *interoceptive overfitting* driven by hyper-precise threat priors. This top-down algorithmic dysfunction synthesizes authentic suffering and triggers descending autonomic cascades that physically perturb the gut. The convergence of high-resolution gastric mapping (BSGM/FlexEGG) and Ecological Momentary Assessment offers a revolutionary objective coordinate system to pinpoint these computational mismatches. Moving forward, the development of closed-loop computational biofeedback DTx promises to directly rewrite the brain’s maladaptive prediction engines through targeted extinction learning. While multimodal phenotyping remains essential to account for electromechanical uncoupling, this digitally-driven paradigm shift provides a rigorously scientific, highly actionable blueprint to liberate millions of DGBI patients from decades of therapeutic stagnation.

## Data Availability

The original contributions presented in the study are included in the article/supplementary material. Further inquiries can be directed to the corresponding author.
